# Mutations in *DARS2* result in global dysregulation of mRNA metabolism and splicing

**DOI:** 10.1038/s41598-023-40107-7

**Published:** 2023-08-10

**Authors:** S. Guang, B. M. O’Brien, A. S. Fine, M. Ying, A. Fatemi, C. L. Nemeth

**Affiliations:** 1 Moser Center for Leukodystrophies at Kennedy Krieger, Kennedy Krieger Institute, 707 N Broadway, Baltimore, MD 21205 USA; 2 Department of Neurology, Johns Hopkins University School of Medicine, Baltimore, MD USA; 3 Department of Neurology and Developmental Medicine, Kennedy Krieger Institute, Baltimore, MD USA; 4 Department of Neurology, Kennedy Krieger Institute, Baltimore, MD USA

**Keywords:** Gene expression analysis, Demyelinating diseases, Paediatric neurological disorders

## Abstract

Leukoencephalopathy with brainstem and spinal cord involvement and lactate elevation (LBSL) is a rare neurological disorder caused by the mutations in the *DARS2* gene, which encodes the mitochondrial aspartyl-tRNA synthetase. The objective of this study was to understand the impact of *DARS2* mutations on cell processes through evaluation of LBSL patient stem cell derived cerebral organoids and neurons. We generated human cerebral organoids (hCOs) from induced pluripotent stem cells (iPSCs) of seven LBSL patients and three healthy controls using an unguided protocol. Single cells from 70-day-old hCOs were subjected to SMART-seq2 sequencing and bioinformatic analysis to acquire high-resolution gene and transcript expression datasets. Global gene expression analysis demonstrated dysregulation of a number of genes involved in mRNA metabolism and splicing processes within LBSL hCOs. Importantly, there were distinct and divergent gene expression profiles based on the nature of the *DARS2* mutation. At the transcript level, pervasive differential transcript usage and differential spliced exon events that are involved in protein translation and metabolism were identified in LBSL hCOs. Single-cell analysis of *DARS2* (exon 3) showed that some LBSL cells exclusively express transcripts lacking exon 3, indicating that not all LBSL cells can benefit from the “leaky” nature common to splice site mutations. At the gene- and transcript-level, we uncovered that dysregulated RNA splicing, protein translation and metabolism may underlie at least some of the pathophysiological mechanisms in LBSL. To confirm hCO findings, iPSC-derived neurons (iNs) were generated by overexpressing Neurogenin 2 using lentiviral vector to study neuronal growth, splicing of *DARS2* exon 3 and DARS2 protein expression. Live cell imaging revealed neuronal growth defects of LBSL iNs, which was consistent with the finding of downregulated expression of genes related to neuronal differentiation in LBSL hCOs. DARS2 protein was downregulated in iNs compared to iPSCs, caused by increased exclusion of exon 3. The scope and complexity of our data imply that DARS2 is potentially involved in transcription regulation beyond its canonical role of aminoacylation. Nevertheless, our work highlights transcript-level dysregulation as a critical, and relatively unexplored, mechanism linking genetic data with neurodegenerative disorders.

## Introduction

DARS2-related leukoencephalopathy, also called Leukoencephalopathy with Brainstem and Spinal cord involvement and Lactate elevation (LBSL), is a rare neurological disorder with a wide phenotypic spectrum. Classic LBSL is characterized by childhood- or juvenile-onset slowly progressive spasticity, cerebellar ataxia and dysfunction in the dorsal column^1^. Severe antenatal and early infantile-onset forms with profound microcephaly and early demise, as well as milder adult-onset cases are also reported^2–4^. LBSL is caused by mutations in the *DARS2* gene, which encodes the mitochondrial aspartyl-tRNA synthetase (mt-AspRS)^5^. mt-AspRS is synthesized in the cytosol and transported to mitochondria where it is responsible for attaching aspartate to its corresponding tRNA.

Nearly all patients carry compound heterozygous mutations in *DARS2*, of which, one is most frequently a splice site mutation in intron 2, causing exon 3 (67 bp) skipping, leading to a frameshift and premature termination codon. These abnormally spliced transcripts are degraded by nonsense mediated decay (NMD); however, van Berge et al. applied a splicing reporter minigene assay and found these mutations to be ‘leaky’, such that full-length transcripts and functional proteins may still be produced^6^. Interestingly, this study also showed that splicing efficiency decreased in cells of a neural lineage in both patients and healthy controls, and to a greater extent in the presence of a *DARS2* mutation. Affected individuals carry either a missense, nonsense, deletion or splice site mutation on the second allele. According to MiSynPat as of June 2023, there are 54 missense and nonsense mutations reported in LBSL patients^7^. A subset of missense mutations dispersed in locations of the N-terminal anticodon-binding domain (R58G, T136S and C152F), the catalytic domain (Q184K, R263Q and D560V) and the C-terminal extension (L613F and L626Q/V) have been studied extensively regarding aminoacylation activity, localization, protein architecture, and solubility^8–10^. In vitro enzymatic assays of mutant proteins showed that only R263Q, D560V and L626Q mutants significantly impaired aminoacylation activity. After being transfected into HEK-293 T cells, C152F, Q184K and D560V mainly affected the expression of mt-AspRS; however, none of these mutations cause gross 3D-structural perturbations or aberrant subcellular localizations of mt-AspRS. Altogether, housekeeping aminoacylation seems not to be the major target of these mutations, pointing to the possibility that mt-AspRS moonlights in the cells by performing non-canonical functions, as has been reported for several cytosolic aminoacyl-tRNA synthetases^11,12^.

The LBSL case study with the largest sample size showed that there was no relationship between the location of the second mutation and disease severity in patients with the common intron 2 splice site mutation, but patients with the combination of mutations in introns 2 and 5 had a milder phenotype^1^. Another case series reported 15 early-onset LBSL patients with profound cortical and white matter dysplasia, of which, 11 patients carried the combination of two missense mutations^3^, suggesting that there may be a genotype–phenotype correlation for LBSL patients.

To date, there have been various conditional *Dars2* knock-out mouse models for studying LBSL pathogenesis. *Dars2* is indispensable for early embryonic development as demonstrated by embryonic lethality of complete knock-out mice^13^. Neuronal ablation of *Dars2* in mice results in severe and progressive phenotypes including hyperactivity, concurrent degeneration in cortex, hippocampus and corpus callosum due to neuronal cell loss, and rampant neuroinflammation; however, *Dars2* depletion in myelin-producing cells does not affect the number of oligodendrocytes or myelin production^14,15^. Combined with long tract involvement in patients, LBSL is considered as a primary axonal disease with secondary demyelination.

The advent of stem cell technology has opened a platform to study phenotypes and mechanisms of neurodegenerative disorders. In the past decade, human induced pluripotent stem cells (iPSCs) have been widely studied and used to construct 3D neural tissues, called human cerebral organoids (hCOs) to better recapitulate the cytoarchitectures of the developing brain. The self-assembly properties of hCOs can be harnessed to establish multiple cell identities under the appropriate culture system and timed application of components^16,17^. With the aid of rapidly evolving single-cell RNA sequencing (scRNA-seq) and computational analysis tools, cellular heterogeneity of hCOs can be dissected at an unprecedented resolution.

In this study we generated hCOs for seven LBSL patients and three healthy controls and performed scRNA-seq (SMART-seq2) on all samples with the hypothesis that there may be non-canonical functions of DARS2 that may be altered in LBSL. We discovered dysregulated expression of genes that encode RNA binding proteins (RBPs) and spliceosomal proteins in LBSL cells, which was exacerbated in neuronal cells. Furthermore, we found pervasive differential transcript usage and alternative splicing events of genes important to RNA and protein binding. This work suggests that dysregulated transcript usage and splicing may underlie at least some of the pathophysiological mechanisms in LBSL.

## Materials and methods

### Maintenance of human iPSCs and generation of hCOs

Peripheral blood mononuclear cells (PBMCs) were isolated from patient whole blood collected from healthy controls and LBSL patients seen at the Kennedy Krieger Institute between 2017 and 2020 (protocol approved by the Johns Hopkins Medical Institutional Review Boards NM_00068613) after obtaining written informed consent from the subject and/or legal guardian. All methods were in accordance with guidelines set forth by Johns Hopkins University and the World Medical Association Declaration of Helsinki. PBMCs were shipped frozen to the Cedars Sinai Induced Pluripotent Stem Cell Core where iPSCs were generated using non-integrating plasmids which rely on episomal expression of reprogramming factors. Additional healthy control lines were obtained from the core and full characterization, immunostaining of pluripotency markers, and karyotyping were completed for all lines. Patient lines were sequenced to confirm the presence of original *DARS2* mutations.

iPSCs were cultured on culture plates coated with Growth Factor Reduced Matrigel (Corning; Corning, NY) and were fed every other day with mTeSR Plus (STEMCELL Technologies; Vancouver, Canada). iPSCs were passaged at 70–90% confluency with ReLeSR (STEMCELL Technologies) per manufacturer’s instructions. hCOs were generated using the STEMdiff™ Cerebral Organoid Kit (STEMCELL Technologies) according to manufacturer’s instructions with modifications. Briefly, iPSCs were seeded in embryoid body (EB) formation medium supplemented with 1 μg/mL ROCK inhibitor (STEMCELL Technologies) on 96-well round-bottom ultra-low attachment plates (Corning). EBs were fed with EB formation medium every other day and were transferred into induction medium in 24-well ultra-low attachment plates on day 5 (one organoid per well; Corning). Organoids were embedded in Matrigel droplets on day 7 and were cultured in the expansion medium on 6-well ultra-low attachment plates (6–8 organoids per well; Corning). After 3 days of stationary culture, organoids were put in the maturation medium on an orbital shaker. To promote oligodendroglial lineage development^18^, beginning at day 28, maturation medium was supplemented with 10 ng/mL platelet-derived growth factor AA (PDGF-AA; PEPROTECH; Cranbury, NJ) and 10 ng/mL insulin-like growth factor 1 (IGF-1; PEPROTECH) for 12 days. Next, on day 40, 40 ng/mL 3,3’,5-Triiodo-L-thyronine (T3; Sigma-Aldrich; St. Louis, MO) was added to maturation medium for another 12 days. The medium was replaced every 3 days throughout the maturation process of organoids until downstream analysis.

### Immunocytochemistry

For immunohistochemical analysis, hCOs were fixed with 4% ice-cold paraformaldehyde at 4 °C for 1 h. Fixed hCOs were embedded in Optimal Cutting Temperature (OCT) Compound and sectioned at 20 μm. Sections were blocked for 1 h in PBS containing 0.3% Triton X-100 and 10% normal goat serum. The sections were incubated at 4 °C overnight with primary antibodies in blocking solution, as follows: rabbit anti-SOX2 (1:300; Cell Signaling Technology, Danvers, MA), rabbit anti-TUJ1 (Alexa Fluor^®^-488 Conjugate; 1:500; MilliporeSigma, Burlington, MA), chicken anti-MAP2 (1:1000; Abcam, Waltham, MA), rabbit anti-GFAP (1:2000; Dako, Santa Clara, CA), rabbit anti-FOXP2 (1:1000; Abcam), rabbit anti-OLIG2 (1:300; MilliporeSigma), chicken anti-MBP (1:2000; ThermoFisher Scientific, Waltham, MA). All secondary antibodies were ThermoFisher Scientific Alexa Fluor™-conjugated secondary antibodies used at a dilution of 1:500. The sections were imaged with Zeiss Axio Imager.M2.

### Organoid dissociation and SMART-Seq2

For RNA sequencing, 70-day old hCOs were washed with ice-cold DPBS and cut into small pieces using a scalpel. Tissue was resuspended in Accutase (Innovative Cell Technologies, San Diego, CA) containing 1 mg/ml of DNase I (Roche, South San Francisco, CA) and incubated at 37 °C for 25 min. Tissue suspensions were mechanically triturated every 5 min and passed through 70 and 30 μm cell strainers. Cell concentration and viability were assessed by Trypan blue staining. Single cell suspensions were cryopreserved in CryoStor^®^ CS10 (STEMCELL Technologies) at − 140 °C for no more than 3 months.

Before thawing single cell suspensions, 1X reaction buffer was made by diluting 10X Lysis Buffer (Takara Bio USA, San Jose, CA) and adding Recombinant RNase Inhibitor (Takara Bio USA) by 3 μL per plate volume. A total of 0.7 μL of 1X reaction buffer was dispensed into each well of a 96-well PCR plate (Fisher Scientific). Single cell suspensions were thawed in warm media and went through 30 μm cell strainers to remove clumps. Single cell suspensions were incubated on ice for 30 min with a live/dead stain and single cells were isolated into PCR plates containing 1X reaction buffer (one plate per cell line) using Fluorescence-Activated Cell Sorting Aria IIu Cell Sorter at the Ross Flow Cytometry Core of Johns Hopkins University. The PCR plates containing sorted cells were kept frozen at -80 ℃ and were shipped on dry ice to MedGenome Inc. (Foster City, CA). Construction of cDNA libraries was done by SMART-Seq v4 Ultra Low Input RNA Kit (Takara Bio USA) and Nextera XT DNA Library Preparation Kit (Illumina, San Diego, CA). Paired-end (100 bp) sequencing was performed using Novaseq 6000 system.

### Bioinformatic analysis of scRNA-seq data

Human genome index was built from GRCh38 DNA primary assembly fasta file (sourced from Ensembl) using R package *Rsubread* (v2.4.3) with default settings^19^. R package *Seurat* (v4.0.3) was used to create an object to filter out the genes that were expressed in fewer than 5 cells and the cells with fewer than 1000 or more than 4,500,000 features (read fragments) detected. Cells with more than 30% of reads mapped to mitochondrial genes were also excluded, leaving 809 cells and 32,909 genes for downstream analysis (Supplemental Fig. 1). Then, datasets from different cell lines were integrated into an unbatched dataset according to developer’s vignette (https://satijalab.org/seurat/articles/integration_introduction.html)^20^. The *FindNeighbors* and *FindClusters* (resolution = 0.5) functions were used to obtain cell clusters. Then the *DefaultAssay* function was performed to set the data to be processed on RNA slot. Markers of each identified cluster were found by the *FindAllMarkers* function and clusters were annotated based on expression of canonical markers of cell types.

The *FindMarkers* function was carried out on the genes that were detected in a minimum 10% of the cells in each group with “MAST” test to identify differentially expressed genes (DEGs) between LBSL and control cells. The threshold of DEGs for enrichment analysis was set as |avglog2FC|> 0.25 and *p.adj* < 0.05. Overrepresentation test for DEGs was conducted with Panther Classification System (http://www.pantherdb.org/) using the statistical overrepresentation test.

The qualified 809 cells were processed with transcriptomic quantifier *kallisto* (v0.46.2)^21^ to obtain the transcript abundance of each cell. R package *DTUrtle* (v0.8.1)^22^ was used to perform differential transcript usage (DTU) analysis, and *BRIE2* (v2.0.5) was used for differential spliced exon (DSE) analysis between LBSL and control cells.

### Transduction of iPSCs with Ngn2 lentivirus particles and neuronal differentiation

*Ngn2* lentiviral particles were produced using transfer vector pTet-O-Ngn2-puro (gift from the Ying Lab, Kennedy Krieger Institute) and the 2nd Gen. Packing Mix & Lentifectin Combo Pack (abm). iPSCs were transduced with *Ngn2* lentivirus for 24 h. Media was changed and iPSCs were grown to confluency followed by gradual puromycin selection (max 5 μg/mL).

Immediately following selection, iPSCs were passaged as single cell suspensions using Accutase and plated at a density of approximately 15,000 cells/well onto Matrigel-coated 12-well plates in mTeSR Plus supplemented with 1 μg/mL ROCK inhibitor. The next day (day 1), media was replaced with a 1:1 solution of mTeSR Plus and DMEM/F12 (Gibco) with 1X N2 (ThermoFisher), and supplemented with 1 μg/mL Doxycycline (MilliporeSigma) to induce Neurogenin 2 expression. On days 2 and 3, media was replaced with DMEM/F12 plus 1 μg/mL Doxycycline. On day 4, cells were detached with Accutase, tapped, collected, and passed through a 0.70 μm cell strainer to further dissociate cell suspension into single cells. Cells were then pelleted by centrifugation at 1000 RCF for 4 min, resuspended in 1 mL of DMEM/F12/N2 with 1 μg/mL Doxycycline and 5 μg/mL puromycin. Cells were seeded at a density of approximately 600,000 cells/well onto 6-well plates coated with 10 μg/mL poly-D-lysine (MilliporeSigma) and 10 μg/mL laminin (MilliporeSigma). The following day (day 5), media was aspirated and replaced with fresh DMEM/F12/N2 and 1 μg/mL Doxycycline. On day 6, media was half changed and replaced with maturation media consisting of BrainPhys Neuronal Media (STEMCELL Technologies) with 1X B27 supplement (ThermoFisher), 0.1 μM ascorbic acid (MilliporeSigma), 0.2 μM Dibutryly cAMP (STEMCELL Technologies), 10 μM DAPT (Peprotech), 10 ng/mL BDNF (Peprotech) and 10 ng/mL GDNF (Peprotech). Neurons were cultured in maturation media, exchanging 50% of media twice a week until day 21 of differentiation. On day 21, neurons were harvested for downstream analyses.

### Splicing analysis of exon 3 of DARS2 gene in iPSCs and neurons

RNA of iPSCs and neurons from healthy controls and LBSL patients were extracted using QIAGEN RNeasy Mini Kit per manufacturer’s instructions. Reverse transcription of RNA to cDNA was done by iScript Reverse Transcription Supermix (Bio-Rad). To study the skipping pattern of *DARS2* exon 3, primers annealing to the junctions of exons 2, 3 and 4 were designed (Supplemental Table 1). PCR Master Mix (ThermoFisher) was used for polymerase chain reaction (PCR), and SYBR Green qPCR Supermix was used for real-time quantitative PCR (RT-qPCR).

### Western blot of DARS2 protein in iPSCs and neurons

iPSCs and neurons from healthy control and LBSL patients were lysed using RIPA (Radio-Immunoprecipitation Assay) buffer supplemented with 1X Protease and Phosphatase Inhibitor Cocktail (FisherScientific) to extract total protein. Pierce BCA Protein Assay Kit (ThermoScientific) was used to quantify the total protein. Then, cell lysates were run on 4–20% precast polyacrylamide gel (Bio-Rad) and blotted onto PVDF membranes (Bio-Rad). Membranes were blocked in Intercept Blocking Buffer (LI-COR) for 1 h at room temperature. Then, membranes were incubated with primary antibodies to human DARS2 (ThermoFisher, 1:1000) and β-Actin (Cell Signaling Technology, 1:1000) in Intercept Blocking Buffer overnight at 4 °C. After washing with TBS-T, secondary antibodies IRDye 680RD and IRDye 800CW were added for 1 h at room temperature. The immunoreactive bands were imaged with LI-COR Odyssey Imaging System and quantification was done by Image Studio software.

### Live cell imaging of neuronal differentiation

On day 4, neuronal cells were passaged at a density of 3*10^4^ cells/well onto PDL/Laminin coated 96-well IncuCyte Imagelock plates. The differentiation of early neuronal cells was tracked under the mode of Neuro Track Scan Type of IncuCyte S3 Live Cell Imaging System. Cell images were acquired every 1 h. The IncuCyte Neurotrack Module was used for quantifiying neurite outgrowth and branch points. The quantitative data obtained at each time point were imported into GraphPad Prism (v8.0.2) and mixed-effects analysis was performed. Differences were considered statistically significant when *p* < 0.05.

## Results

### Clinical profiles of LBSL patients

We generated iPSCs from seven pediatric LBSL patients (2 female and 5 male, Table 1). According to the previous case study^1^, patients with combinations of mutations in introns 2 and 5 had a milder phenotype, therefore, we divided the cohort in two subgroups: Group 1 patients with only one or no splice site mutations (labeled MIS), and Group 2 patients having splice site mutations in introns 2 and 5 (labeled SPLICE). The mean age of onset was comparable for the two subgroups (MIS: 2.56 ± 1.88 years old, SPLICE: 1.94 ± 2.68 years old). Nevertheless, MIS patients seemed to present higher SARA scale (12.83 ± 1.20 VS. 5.50 ± 1.00) and MRI Loes Scale (13.00 ± 3.24 VS. 5.25 ± 2.25) within shorter follow-up duration (4.56 ± 3.19 years versus 7.06 ± 6.31 years) compared to SPLICE patients. For all, the motor function and manual ability (GMFCS and MACS, respectively) scores were mild, ranging from I to II, and all patients were ambulating and performing daily functions independently at the time of their evaluation. All but one patient reported episodic regression of motor symptoms during a minor illness or fever lasting for the duration of the illness with recovery thereafter.

**Table 1:** Clinical characterizations of included LBSL patients.

LBSL patient		Mutation 1	Mutation 2	Sex	Onset age (year)	Age at follow-up (year)	GMFCS*	MACS^&^	SARA score^#^	MRI loes score^ω^
MIS	1A	c.753G > T (p.M251I)	c.228-20delTTinsC (p.R76Sfs*5)	F	1.5	10	I	I	10.5	10
	1B	c.473A > T (p.E158V)	c.829G > A (p.E277K)	M	0.75	1.5	II	II	N/A	7
	1C	c.265C > T (p.L89F)	c.492 + 2 T > C (p.M134_K165 del)	F	5	10	I	I	14.5	22
	1D	c.228-20delTTinsC (p.R76Sfs*5)	?	M	3	7	I	I	13.5	13
SPLICE	2A	c.228-20delTTinsC (p.R76Sfs*5)	c.492 + 2 T > C (p.M134_K165 del)	M	0.83	13	II	II	UNK	UNK
	2B	c.228-17C > G	c.492 + 2 T > C (p.M134_K165 del)	M	0	9	I	I	6.5	3
	2C	c.228-17C > G	c.492 + 2 T > C (p.M134_K165 del)	M	5	5	I	I	4.5	7.5

*UNK* Unknown because patient is international/not enrolled in ongoing natural history study, *N/A* Not applicable due to age too young to participate in the test.

*Gross Motor Classification Scale (I–V most severe).

^&^Manual Ability Classification Scale (I–V most severe).

^#^Standardized Assessment and Rating of Ataxia Scale (0–40 most severe).

^Ω^Modified Loes Score (0–40 most severe).

### Generation and characterization of human cerebral organoids

**Figure 1:**
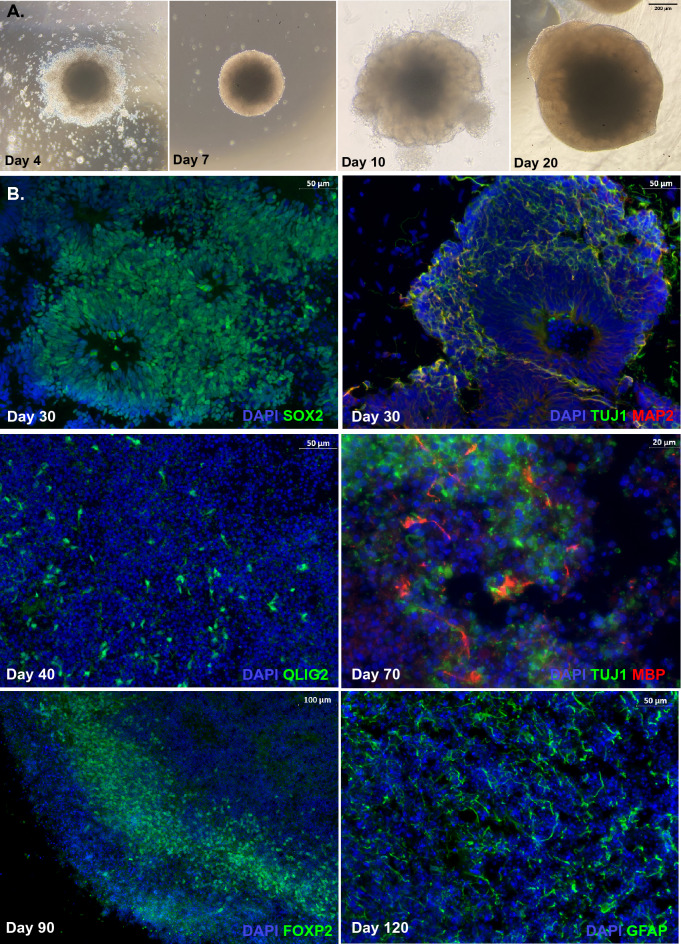
Representative images from a Control human cerebral organoid (hCO). (**A**) Morphological changes of hCO under light microscope. By day 4 after differentiation, an embryoid body (EBs) with round and smooth edges form in the EB formation medium. By day 7, the EB develops brighter borders in the induction medium. The organoid embedded in Matrigel droplet shows budding of the surface around day 10. Organoids kept in the maturation medium exhibit dense core and optically translucent edges by day 20 and beyond. (**B**) Immunostaining of human cerebral organoids during development. Prototypical SOX2 + ventricular zone (VZ) at day 30, while TUJ1 + MAP2 + neurons migrate to the outer layer of VZ. By day 40, OLIG2 + OPCs expand after treatment of PDGF-AA and IGF1 for 12 days. OPCs are induced to MBP producing oligodendrocytes by T3 treatment. FOXP2 + cells form a layer along the organoid. GFAP + astrocytes are well-developed after long-term (120 days) maturation.

Generation of hCOs included four periods: embryoid body formation (Day 0–5), induction (Day 5–7), expansion (Day 7–10) and organoid maturation (after Day 10; representative Control hCO shown in Fig. 1A). iPSCs that were seeded on ultralow-attachment plates self-organized into 3D embryoid bodies (EBs), and round and smooth edges should be visible before proceeding to the induction stage. After induction, the borders of EBs turned brighter indicating neuroectodermal differentiation at which point organoids were embedded in Matrigel droplets to promote the expanding of neuroepithelium, presenting as budding on the surface. Organoids in the maturation medium continued to develop and mature for over 2 months. LBSL organoids developed with no signs of growth difficulties compared to control and the overall gross structure were comparable. Immunostaining revealed the presence of prototypical structures of ventricular zones (VZs) with SOX2 + radial glial cells in hCOs at the early stage (representative Control hCO shown in Fig. 1B, Day 30), and TUJ1 + and MAP2 + post-mitotic neurons migrated to the outer layer of subventricular zones (SVZs; Day 30). Subsequent treatment of hCOs with platelet-derived growth factor AA (PDGF-AA) and insulin-like growth factor 1 (IGF-1) promoted the expansion of oligodendrocyte progenitors (OPCs; Olig2, Day 40), followed by the treatment of thyroid hormone (T3) which induced OPCs to differentiate into myelin basic protein-producing oligodendrocytes (Day 70). With the extension of maturation, FOXP2, the cortical layer VI marker, was also expressed around the superficial layer of hCOs and GFAP + astrocytes were also observed (Day 90).

### Cellular diversity of human cerebral organoids revealed by scRNA-seq

LBSL and control cells were well intermixed without obvious batch effects; unsupervised clustering and visualization using UMAP revealed nine transcriptionally distinct clusters (Supplemental Fig. 2A,B), which were annotated according to the expression of canonical cell-type markers^23,24^. Cluster annotations (Supplemental Fig. 2C) revealed the presence of *MKI67* + *TOP2A* + *CENPF* + proliferative neuroepithelial cells (NECs, cluster 0), *NES* + *PAX6* + radial glial cells (RGCs, cluster 1), *MAP2* + *DCX* + *STMN2* + cortical neuronal cells (CNs, cluster 2), *PIFO* + *FOXJ1* + cilium bearing cells (CBCs, cluster 6), *AQP1* + choroid plexus cells (ChP-AQP1, cluster 7), and *TTR* + *CLDN5* + choroid plexus cells (ChP-CLDN5, cluster 8). For clusters without self-evident markers, GO term enrichment analysis was used to analyze the function of differentially expressed genes (DEGs, *p.adj* < 0.05) found by the *FindAllMarkers* function. Cluster 3 highly expressed genes related to unfolded protein response (UPR) mediated by endoplasmic reticulum (ER), and presented glycolytic signature (*ALDOA* and *PGK1*), reflective of UPR-related cells (UPRCs). DEGs of cluster 4, such as *SLC17A8*, *SYNE2*, *LAMB1*, *SLC4A10*, *FGFR2*, *S100B* and *LGI1*, were enriched to central nervous system development (GO:0007417) and synapse (GO:0045202). Cluster 5 showed high expression of ribosomal protein genes (RPGs).

*DARS2* expression was queried across the different clusters and is shown in Supplemental Fig. 2C. Interestingly, *DARS2* was predominately expressed in NECs but downregulated in all other differentiated post-mitotic cell clusters.

### RNA metabolic process and splicing are dysregulated in LBSL hCOs

**Figure 2:**
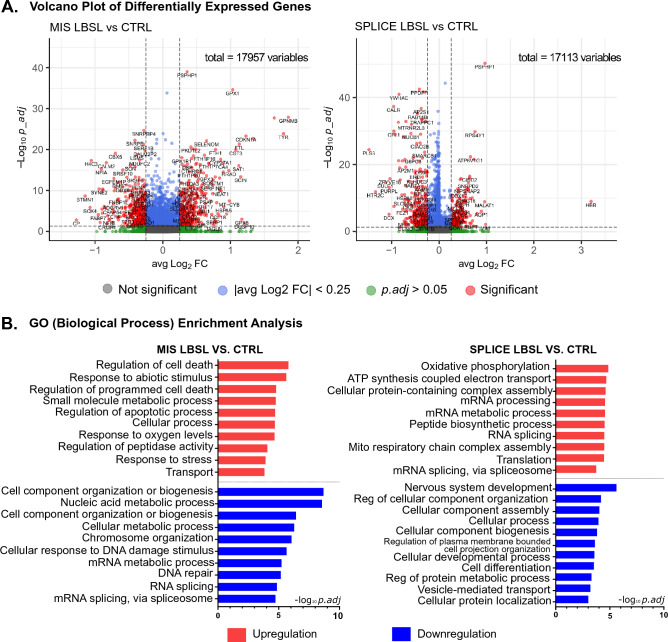
Volcano plot of DEGs. (**A**) MIS LBSL cells versus control cells. SPLICE LBSL cells versus control cells. (**B**) Top 10 enriched GO terms (biological process) by DEGs. MIS LBSL cells versus control cells and SPLICE LBSL cells versus control cells are shown.

**Figure 3:**
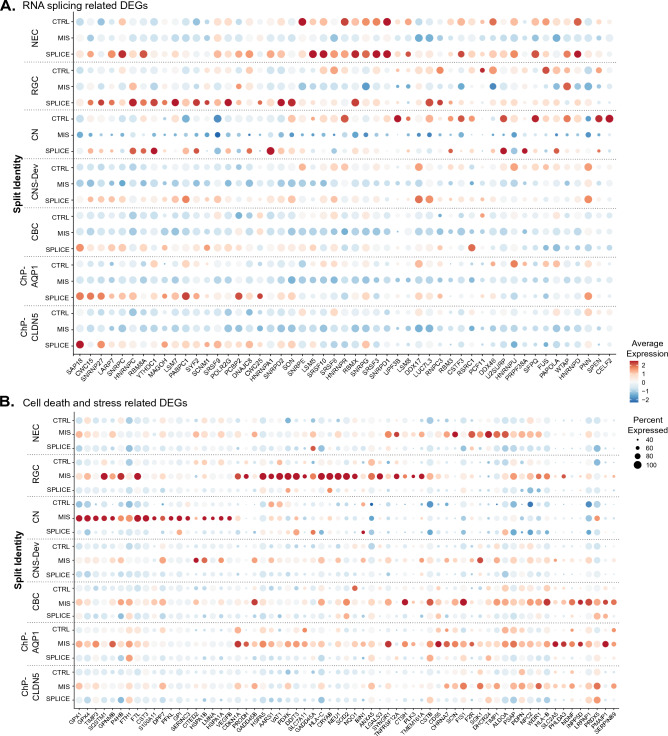
Dot plots of DEGs under the GO terms “RNA splicing” (**A**) and “Regulation of cell death” (**B**).

We identified 736 (333 up, 403 down) and 490 (198 up, 292 down) DEGs in MIS and SPLICE compared to control cells, respectively (volcano plot, Fig. 2A). Intriguingly, gene set enrichment analysis (Fig. 2B) showed several biological processes, such as mRNA metabolic process and splicing, downregulated in MIS cells but upregulated in SPLICE cells. Heatmaps of DEGs under the GO terms “RNA splicing” and “Regulation of cell death” confirmed the unique DEG profiles of two subsets of LBSL hCO cells (Supplemental Fig. 2). Dot plot analysis demonstrated that genes involved in mRNA metabolism and splicing were downregulated in MIS cells with exacerbation in neuronal cells but upregulated in NECs, RGCs and ChP cell clusters in SPLICE LBSL cells (Fig. 3A). Genes involved in regulation of cell death and stress were upregulated mainly in MIS, and different cell lineages upregulated unique key genes relating to cell stress (Fig. 3B). Furthermore, upregulated translation (mainly in NECs and RGCs) and oxidative phosphorylation (mainly in ChP cells) were unique for SPLICE.

### Pervasive differential transcript usage (DTU) events involved in protein translation and metabolism in LBSL hCOs

**Figure 4:**
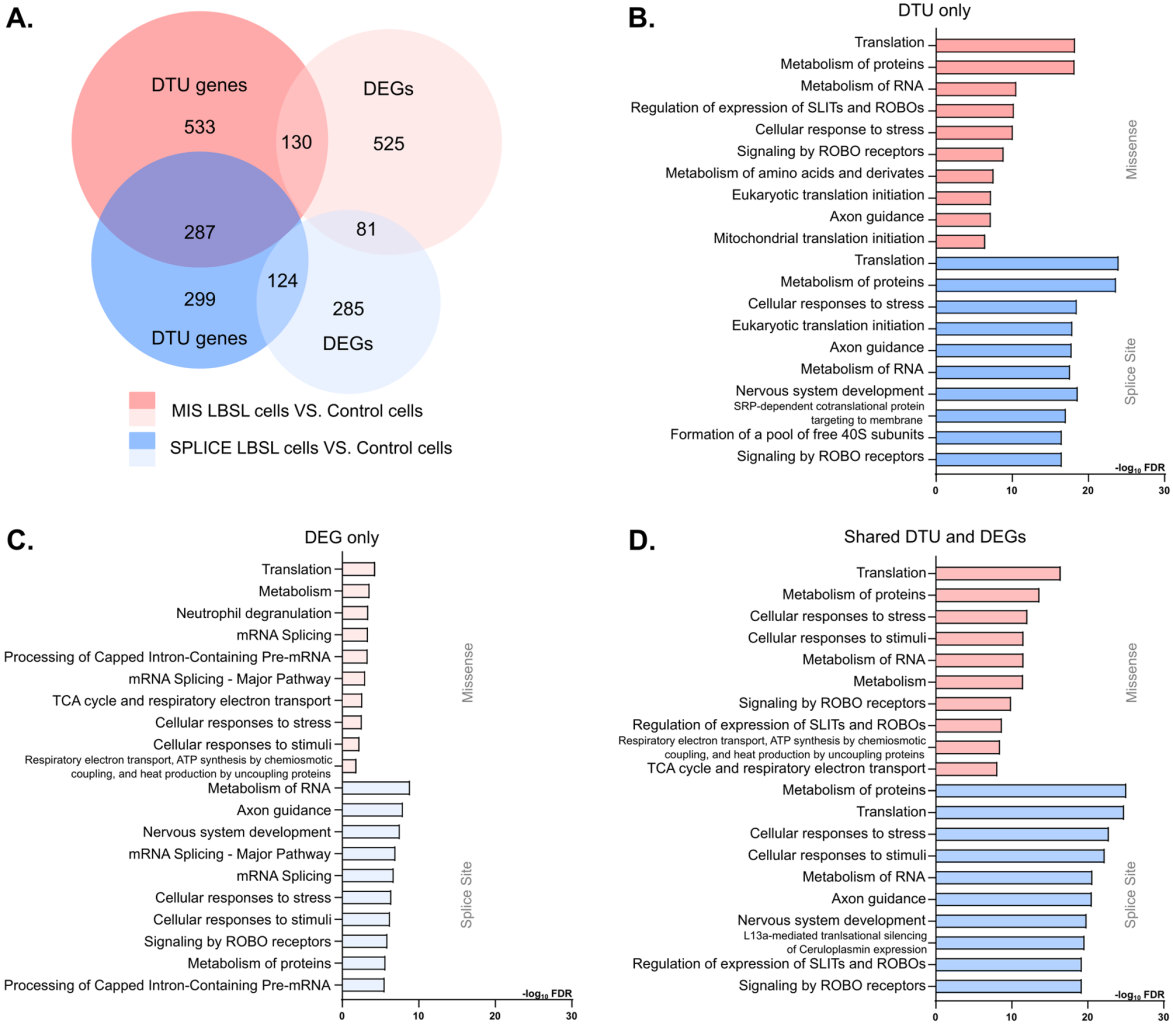
Summary of DTU and DEG analyses. (**A**) Venn diagram of DTU genes and DEGs. (**B**) Reactome enrichment analysis showing the top 10 ranked terms for MIS LBSL cells (red) and SPLICE LBSL cells (blue) for genes with significant DTU events. (**C**) Reactome enrichment analysis for MIS and SPLICE cells for DEGs, and (D) Reactome enrichment analysis for MIS and SPLICE cells significant DTU and DEG cells. Colors in bar plots correspond to Venn shown in A.

The discovery of dysregulated mRNA processing and splicing pathways in LBSL cells led us to investigate transcript-level changes, which cannot be revealed through DGE analysis. Differential transcript usage (DTU) analysis was done by the R package *DTUrtle* (v0.8.1)^22^ in 809 filtered cells. Genes expressed at lower than 5% of the total expression level and in fewer than 5% of the cells of the smallest group were pre-filtered out to maintain a high statistical power, leaving 86,629 and 79,338 transcripts in the comparisons of MIS versus control and SPLICE versus control, respectively. We only studied significant DTU genes (OFDR < 0.01) expressed in at least 60% of cells, with at least one driving transcript identified. Compared to control cells, a total of 950 and 710 genes with DTU events were detected in MIS and SPLICE LBSL hCO cells, respectively (see Venn summary in Fig. 4A and list of DTU genes in Supplemental Table 2).

In Section “RNA metabolic process and splicing are dysregulated in LBSL hCOs”, gene-level analysis identified 736 and 490 DEGs in MIS and SPLICE LBSL cells, respectively, with only 81 DEGs shared between the two groups. Transcript-level analysis found 281 DTU overlapping events between the two groups of LBSL cells (Fig. 4A). Although the two groups of LBSL cells presented some distinct gene-level changes, their transcript-level changes shared more similarity. To better understand the molecular consequences of these DTU events, we analyzed significant findings using Reactome which revealed that genes of both LBSL groups were enriched into similar pathways, such as protein translation and metabolism, cell stress, and neuronal axon development (Fig. 4B).

**Figure 5:**
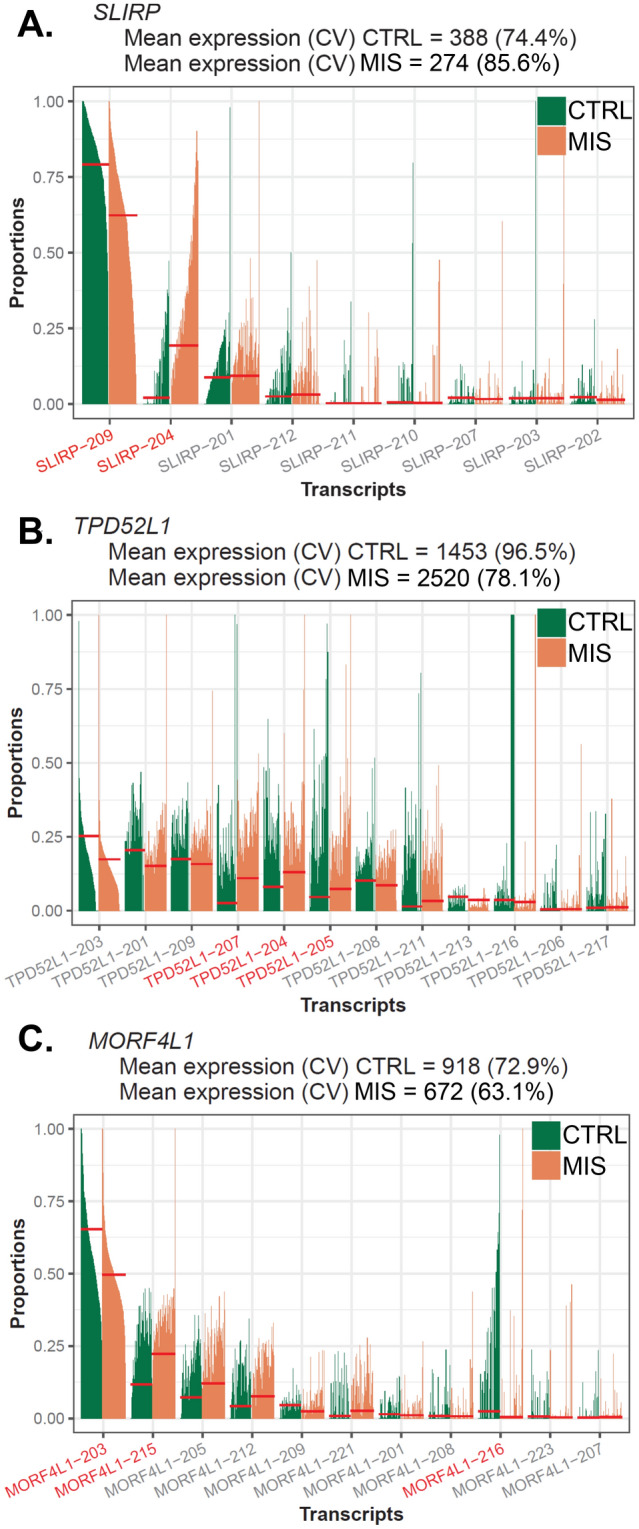
Top 3 significant DTU genes in the comparison of MIS LBSL cells versus control cells. Bar plots of proportions of each transcript per cell (mean proportion fit: short red line). Significant DTU transcripts are represented as red fonts. (**A**) The expression of SLIRP was downregulated in MIS cells, and DTU analysis revealed the switching from protein-coding to nonsense mediated decayed transcripts, indicating that dysregulated splicing may have led to the decreased gene expression. (**B**) DTU analysis further elucidated that LBSL cells not only had higher transcript usage of protein coding transcripts of *TPD52L1,* (*TPD52L1*-204 and *TPD52L1*-207) but also of intron-retaining transcripts (*TPD52L1*-205). (C) Finally, DTU analysis indicated *MORF4L1 s*witching from transcripts with long 3' untranslated region (UTR) to transcripts with short 3’ UTR.

In cross comparing the DTU events with DEGs, we found approximately 85% of DTU genes failed to be detected by the gene-level analysis. Reactome analysis revealed that these ‘DEG only’ genes were mainly related to metabolic process and translation (Fig. 4C) in MIS cells, with more specified pathways, “metabolism of RNA”, “mRNA splicing”, and “nervous system development” noted for SPLICE cells. Within these data, we find transcript-level analysis to have revealed additional genes missed by DGE analysis and further study of the relevance of individual transcripts of one gene, such as expression level and cellular localization, can be carried out based on the findings from DTU analyses. For genes that were detected by DGE and DTU analysis, DTU analysis would still add another layer of complexity (Reactome pathway in Fig. 4D). To illustrate, of the top 3 significant DTU genes (*SLIRP*, *TPD52L1* and *MORF4L1*), we found *SLIRP* to be downregulated in MIS LBSL cells (avg_log2FC = − 0.27, *p.adj* = 1.79E − 08), and DTU analysis showed a switching from protein-coding (*SLIRP*-209) to nonsense mediated decayed transcripts (*SLIRP*-204) in LBSL cells, indicating that dysregulated splicing may have led to decreased gene expression (Fig. 5A). Furthermore, DGE analysis identified *TPD52L1* to be upregulated in MIS LBSL cells (avg_log2FC = 0.78, *p.adj* = 4.31E-14; Fig. 5B), and DTU analysis further elucidated that LBSL cells not only had higher transcript usage of protein coding transcripts (*TPD52L1*-204 and *TPD52L1*-207) but also of intron-retaining transcripts (*TPD52L1*-205). Finally, *MORF4L1* was found to be downregulated in MIS LBSL cells in DGE analysis (avg_log2FC = − 0.30, *p.adj* = 0.025; Fig. 5C), but DTU analysis showed a switching from transcripts with long 3’ untranslated region (UTR) to transcripts with short 3’ UTR.

### Not all LBSL cells benefit from “leaky” expression of DARS2 splice site mutation

**Figure 6:**
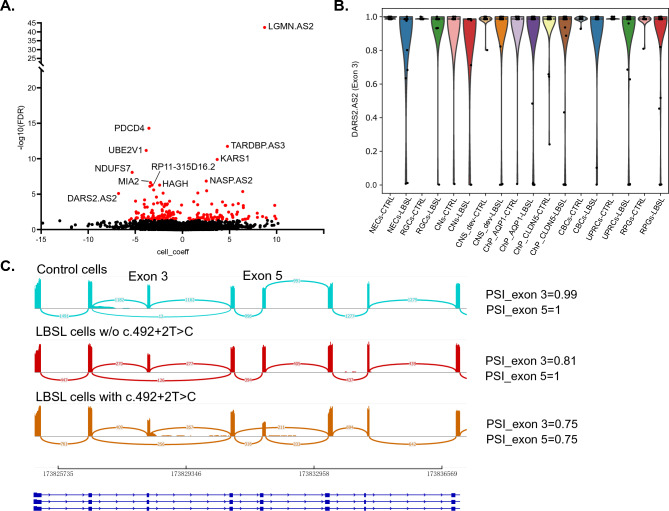
Differentially spliced exon (DSE) analysis by BRIE2 software. (**A**) Volcano plot for DSEs between LBSL and control cells. (**B**) Violin plot on *DARS2* (Exon 3) for estimated PSI between LBSL and controls cells in each cell type. (**C**) Sashimi plot of *DARS2* (Exons 1–8) via merged BAM files, showing the “leaky” nature of mutations in Introns 2 and 5.

When using disease state as a cell-level feature, *BRIE2* detected 194 genes with DSEs (FDR < 0.05) associated with LBSL, as shown in Fig. 6A and listed in Supplemental Table 3 (top 20 genes). Gene set enrichment analysis of DSE genes revealed a similar enrichment as DTU analysis: DSE genes mainly functioned in translation regulator activity (GO: 0045182; *MIF4GD*, *EEF1D*, *MTIF2*, *EIF4B, EIF2AK2*, *EFL1*, *MTIF3*, *EIF2B3*, *EIF2AK4*), RNA binding (GO:0003723; *PDCD4*, *TARDBP*, *KARS1*, *SRP72*, *DARS2*, *ZCCHC17*, *PPHLN1*, *SAFB*, *RNMT*, etc.) and protein binding (GO:0005515; *LGMN*, *UBE2V1*, *NDUFS7*, *NASP*, *MIA2*, *HAGH*, *FNTA*, *GALNT10*, *COX7B*, etc.).

Interestingly, *DARS2* (exon 3, FDR = 7.986E − 06) was identified as a DSE; however, the total cell number was too small to draw conclusions about splicing differences among the different cell identities (Fig. 6B). Although transcripts lacking exon 3 were expressed in control cells, they usually co-existed with normal transcripts (PSI ≠ 0); in contrast, some LBSL cells expressed only abnormally spliced transcripts (PSI = 0) indicating that not all LBSL cells benefit from the “leaky” mechanism of the splice site mutation. Visualization of merged BAM files via sashimi plot verified that the PSI of exon 3 of *DARS2* in LBSL cells was lower than that of control cells. We also observed c.492 + 2 T > C, which is located in intron 5, caused exon 5 skipping in a similar “leaky” fashion (Fig. 6C).

**Figure 7:**
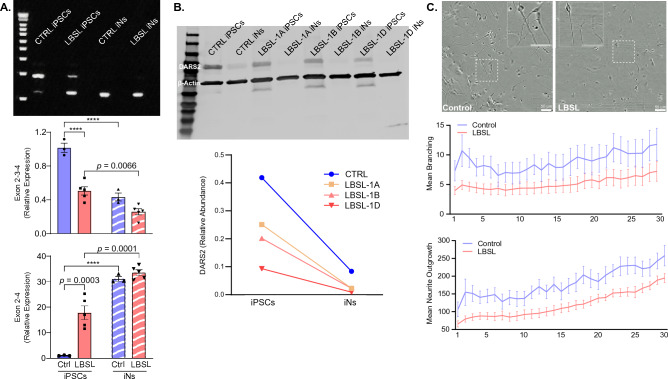
(**A**) Splicing analysis of exon 3 of *DARS2* gene include agarose electrophoresis of PCR products and RT-qPCR results. (**B**) Western blot of DARS2 protein in iPSCs and iNs. (**C**) IncuCyte live cell imaging of early neuronal cells.

Multiple primers annealing to junctions of exons 2, 3 and 4 of *DARS2* gene were designed to explore the skipping pattern of exon 3 in iPSCs and iNs. Agarose electrophoresis of PCR products (Fig. 7A) revealed that a small amount of abnormally spliced transcripts excluding exon 3 existed in control iPSCs, and the ratio of normal to abnormal transcripts was greater than 1; however, the ratio shifted in LBSL iPSCs, consistent with scRNA-seq data. When iPSCs were differentiated into iNs, transcripts excluding exon 3 became predominant regardless of disease status. RT-qPCR results demonstrated that the expression level of transcripts containing exon 3 in LBSL iPSCs was lower than that in control iPSCs (*p.adj* < 0.0001), and the level of transcripts excluding exon 3 in LBSL iPSCs was 20-fold higher than that in control iPSCs (*p.adj* = 0.0003). With the differentiation of iPSCs into iNs, transcripts containing exon 3 were significantly downregulated while transcripts lacking exon 3 increased.

### DARS2 is downregulated in post-mitotic neurons

Data across cell clusters shown in Supplemental Fig. 2 (Section “Cellular diversity of human cerebral organoids revealed by scRNA-seq”) illustrate that *DARS2* was predominately expressed in NECs but downregulated in the differentiated post-mitotic cell clusters. Because it would be difficult to isolate NECs or neuronal cells from hCOs for downstream expression analysis we established iPSC cell lines with *Ngn2* integrated into the genome by lentivirus transduction to rapidly and efficiently differentiate neuronal cells. Western blot of DARS2 demonstrated that DARS2 is highly expressed in iPSCs but downregulated in iPSC-derived neurons (iNs) in both control and LBSL cells, consistent with scRNA-seq data (Fig. 7B).

### Neuronal growth deficits in LBSL

Although two groups of LBSL hCO cells presented unique DEG profiles when compared to control cells, common to both of these groups were downregulation of genes related to CNS development and neuronal differentiation (Supplemental Fig. 4). Live cell imaging was applied to monitor the differentiation process of early iNs (Fig. 7C), revealing reduced neurite outgrowth in LBSL iNs compared to control (*p* = 0.016) and a trend suggesting LBSL iNs had fewer branch points than control iNs.

## Discussion

Since 2007, more than 200 LBSL cases have been reported globally, with patients presenting varied ages of onset and disease severities. Little progress has been made towards the understanding of LBSL’s pathogenic mechanism and mouse models cannot recapitulate the exact nature of LBSL mutations limiting their utility. As embryonic brain tissue is of course inaccessible, hCOs provide an unprecedented way to investigate neurodevelopment and related disorders. Compared to pure neuronal populations produced in 2D culture, the sophisticated 3D structures of hCOs recapitulate vital cytoarchitectures of the developing brain, such as interactive dynamics of multiple cell lineages and complex neural circuits. Broadly, current methodologies for induction of hCOs from hPSCs can be classified into two categories: unguided methods that are dependent on self-organization and development of hPSCs under proper culture conditions^16,17^, and guided methods that utilize small molecules and growth factors to produce brain region-specific organoids^25–28^. Early oligodendrocyte progenitor cells (OPCs) are generated from ventral forebrain and migrate to dorsal forebrain, so oligodendroglial lineages were only identified in Quadrato’s unguided hCOs^29^ after long-term culture but absent in most early-stage unguided hCOs^30^. In our study, we adopted Madhavan’s protocol, in which OPCs and myelinating oligodendrocytes (OLs) are induced by means of timed exposure to PDGF-AA, IGF1 and T3^31^; with this method, however, myelinating OLs are mostly along the edges of hCOs (Fig. 2B), perhaps due to limited penetration^31,32^. SMART-seq2, as a low-throughput method unfortunately has no advantage in detecting non-mainstream cell types, and consequently OLs and astrocytes were absent from our dataset of 70-day-old hCOs which is a weakness of this study.

The major technical hurdle of organoid models is the insufficient diffusion of oxygen and nutrients to the innermost regions of hCOs, creating a necrotic core over long-term culture. These organoid systems show a preferential expression of genes related to glycolysis and ER stress and cell stress might compromise the subtype specification of cell types^30,33^. Similarly, we identified a cluster of cells with glycolytic signature (cluster 3, UPRCs), which were also reported as a neuronal subset in hCO models from other labs^23,34^. However, Tanaka’s synthetic analyses of published scRNA-seq data of hCOs generated by multiple labs via different protocols showed there was no enrichment of genes involved in the given neuropsychiatric or related disorders, indicating the current organoid systems are applicable in modeling these disorders^30,35,36^.

Transgenic mouse models with *Dars2* or *Wars2* gene deletion prompt that loss of mitochondrial aminoacyl-tRNA synthetases (mt-aaRSs) could cause impairment of mitochondrial protein synthesis and disruption of protein homeostasis, leading to activation of integrated stress response (ISR)^13,37^. During cell stress, stress granules (SGs), composed of a series of RNA binding proteins (RBPs), translation initiation factors, 40S ribosomal subunits and mRNA, assemble to cope with the crisis by arresting global translation and regulating mRNA expression—thus affecting cell signaling and apoptosis^38–40^. When cell stress lasts for a long duration, SGs are retained in the cytoplasm, leading to dysregulation of RBP components. Our scRNA-seq data also revealed upregulation of cell stress and apoptosis related genes and downregulation of RBP genes in MIS LBSL cells, where at least one missense mutation is present. The cell stress phenotype from patients with only splice site mutations (SPLICE) LBSL cells was milder, and genes involved in oxidative phosphorylation, translation, and RBPs were upregulated, which could be compensatory. To date, there is no consistent evidence of major losses in enzyme activity or of structural perturbations caused by *DARS2* missense mutations. Additionally, the splice site mutations in introns 2 and 5 are “leaky” so full-length transcripts and functional proteins can be produced in LBSL patients. Furthermore, no correlation was found between disease severity and residual enzyme activity of mt-AspRS from LBSL patient lymphoblasts^1^. Housekeeping aminoacylation seems not to be the major target of these mutations, pointing to the possibility that mt-AspRS moonlights in the cells by performing non-canonical functions such as angiogenesis, immune response, tumorigenesis or neurodevelopment, as has been reported for several other aminoacyl-tRNA synthetases^41,42^. Therefore, the possibility that mt-AspRS is directly involved into the regulation of RBPs cannot be ruled out.

For neuronal cells with cellular polarity, RBPs participant in transporting mRNA to axon terminals for local protein synthesis, which plays a crucial role in neuronal differentiation and function. Our study found that genes involved in CNS development and neuronal differentiation were downregulated in both LBSL groups, and we followed up by examining these processes in iPSC-derived neurons using live and long-term cell imaging, only to uncover growth deficits in LBSL neurons. Importantly, oligodendroglial differentiation and myelin formation are highly dependent on neuron-derived growth factors, neuronal firing activity and physical contact between neuronal and oligodendroglial cells, thus growth deficits within neurons can lead to secondary white matter lesions^43^.

mRNA metabolism and splicing are found to be dysregulated within a growing number of neurological disorders^38,44,45^, and although altered within LBSL cells compared to control, their precise role in disease pathogenesis is unclear. To further investigate, we expanded our analyses to transcript-level changes, which are overlooked by DGE analysis, and identified pervasive DTU events in LBSL cells, most of which did not overlap with DEGs. The variance between gene expression and transcript usage may result from antagonistic expression changes in multiple transcripts of one gene which cancels out the net change of gene expression, or that DTU event only occurs in lowly-expressed transcripts^46^. There is an emerging perspective that compared to gene-level changes, transcript-level alterations provide a more specific disease signature^47^. Although LBSL MIS and SPLICE cells present a rather different picture of dysregulated genes, the consequences at the transcript level are more converged, which involve protein translation and metabolism, cell stress and axonal differentiation.

The ISR can be activated by four kinases (PERK, GCN2, PKR and HRI) to maintain protein homeostasis when cells encounter stresses such as mitochondrial dysfunction, oxidative stress, unfolded protein response and nutrition deprivation. The above four kinases inhibit the eukaryotic translation initiation factor eIF2B by phosphorylating eIF2α to inhibit global protein translation while inducing expression of ATF4 and DDIT3. As a result, ISR activation has extensive downstream effects on the expression of genes related to biosynthesis, aminoacyl-tRNA synthetases, translation factors and proapoptosis^48,49^. Long-term activation of the ISR has been associated with various neurological disorders. In our study, gene-level analysis found a number of upregulated genes related to cell stress and apoptosis (including *DDIT3*) in LBSL cells, but it failed to detect the expression changes of ISR kinase genes. DTU analysis, however, found both MIS and SPLICE LBSL cells had abnormal transcript usage of *EIF2AK1*. Looking more closely, LBSL cells showed a higher transcript usage of the protein-coding transcript (*EIF2AK1*-201) than control cells, whereas, controls cells had higher usage of the nonsense mediated decay transcript (*EIF2AK1*-203). Overall, this may suggest increased EIF2AK1 protein (HRI kinase) in LBSL cells and activation of the ISR. Of note, DTU analyses also detected a series of DTU events belonging to translation initiation and elongation factors (*EIF3C*, *EIF3E*, *EIF3F*, *EIF3I*, *EIF3L*, *EIF4A2*, *EIF4B*, *EIF4E*, *EIF4H*, *EEF1B2, EEF1D* and *EEF2*), as well as genes of proteosome family (*PSMA3*, *PSMA4*, *PSMB3*, *PSMC4*, *PSMD6*, *PSME2* and *PDMG2*) in LBSL cells. DTU events in LBSL may have important implications on protein function as switching between protein-coding and non-coding transcripts may affect protein totals, and switching among protein-coding transcripts may change the ratio of multiple protein isoforms with different biological functions and/or subcellular localizations. Increasing evidence supports dysfunctional protein translation and metabolism to contribute at least in part to several neurodevelopmental and neurodegenerative disorders.

Transcript-level quantifications are done by imputation from short-read sequencing data indexed by existing genomic annotations. Consequently, DTU analysis is hindered by incomplete annotations and by the nature of short-read sequencing. To better understand transcript expression within our samples, we performed alternative splicing analysis on cassette exons. With *BRIE2*, *DARS2* (exon 3) was identified as one of the DSEs associated with LBSL, a finding verified by PCR and RT-qPCR which shows that exon 3 exclusion was significantly increased in LBSL cells. In these experiments, transcripts lacking exon 3 became predominant after iPSCs differentiated into iNs, a finding consistent with the observed downregulation of DARS2 protein western blots. These data suggest DARS2 is highly expressed in stem cells and plays a more important role during differentiation, which may explain the embryonic lethality of complete *Dars2* knock-out mice.

Although it is well accepted that splice site mutations within intron 2 of *DARS2* are “leaky”, we revealed, for the first time, that at the single-cell level, some LBSL cells only expressed transcripts lacking exon 3 (PSI = 0), indicating that not all LBSL cells were capable of the “leaky” full length production of DARS2. We also demonstrated that the rather common c.492 + 2 T > C mutation could cause exon 5 skipping, and that this mutation is also leaky, resulting in some degree of full-length transcripts. Based on our findings at the time point examined, even in LBSL patients with the same mutations, the dominant *DARS2* transcripts and final DARS2 protein level expressed in cells may be random, resulting in phenotype differences among cells and individuals.

## Conclusion

The scope and complexity of our data do not immediately lend themselves to simple mechanistic reduction of LBSL. Nevertheless, our work highlights transcript-level dysregulation as a critical, and relatively unexplored, mechanism linking genetic data with neurodegenerative disorders. At gene- and transcript-level analyses, we revealed that dysregulated RNA and protein metabolism, splicing and translation may underlie at least some of the pathophysiological mechanisms in LBSL, and that this may serve as a starting point for further investigations.

### Supplementary Information

Supplementary Information.

## Data Availability

The datasets generated and/or analyzed during the current study are available from the corresponding author on reasonable request and will be made available in NCBI’s Gene Expression Omnibus (GEO) upon publication.
