# Supplementary material for: Mutations in DARS2 result in global dysregulation of mRNA metabolism and splicing
Source: Sci Rep. 2023 Aug 10;13:13042. doi: 10.1038/s41598-023-40107-7 (PMC10415389; doi:10.1038/s41598-023-40107-7)
Supplement: Supplementary file 1 — Supplementary Information. [file 41598_2023_40107_MOESM1_ESM.pdf]

SUPPLEMENTAL DATA

Mutations in *DARS2* result in global dysregulation of mRNA metabolism and splicing

Guang S[1,2], O'Brien BM[1], Fine AS[3], Ying, M[2,4], Fatemi A[1,2], Nemeth CL[1,2*]

[1]Moser Center for Leukodystrophies at Kennedy Krieger, Kennedy Krieger Institute, Baltimore, MD, USA;
[2]Department of Neurology, Johns Hopkins University School of Medicine, Baltimore, MD, USA;
[3]Department of Neurology and Developmental Medicine, Kennedy Krieger Institute, Baltimore, MD, USA
[4]Department of Neurology, Kennedy Krieger Institute, Baltimore, MD, USA

*Corresponding Author:

Christina Nemeth Mertz, PhD
Kennedy Krieger Institute
707 N Broadway
Baltimore, MD 21205
667-205-4288 (p)
443-923-2695 (f)
Mertz@kennedykrieger.org

Supplemental Table 1. Primers used for DARS2 exon 3 inclusion/exclusion

Experiments	Primers	Primer sequence (5'-3')	Primer length (bp)
PCR	E2A-fwd	GTGGAGAGTTGCGTTCGTCT	20
	E4-rev	AGGACGGGAAATGACTGTACC	21
RT-qPCR (Transcripts with exon 3)	E2A-fwd	GTGGAGAGTTGCGTTCGTCT	20
	E3_4-rev	TGAACAAGCCCATCGAAATCTC	22
RT-qPCR (Transcripts without exon 3)	E2B-fwd	CCAACACATGTGGAGAGTTGC	21
	E2_4-rev	TTCAGTACCGAAGTCGGCAG	20
RT-qPCR (Housekeeping genes)	PPIA-fwd	GTCAACCCCACCGTGTTCT	19
	PPIA-rev	CTGCAAACAGCTCAAAGGAGAC	22
	PSMB4-fwd	CGTCCACTCCCGATTCCTTC	20
	PSMB4-rev	AATGCGAGAGATGTTGCGGA	20

Supplemental Table 2. Top 20 significant DTU genes in two groups of LBSL cells

Gene	# Transcripts Detected	# Sig Transcripts	Gene Overall FDR	Min Transcript OFDR	Max DTU Difference	Chr	% Cells Expressing
**MIS LBSL cells VS. Control cells**							
SLIRP	9	2	1.85E-78	1.58E-86	0.173	14	0.973
TPD52L1	12	3	1.16E-67	1.67E-57	0.085	6	0.905
MORF4L1	11	3	3.51E-57	1.16E-20	-0.158	15	0.991
TRIM22	8	3	6.66E-50	2.10E-38	-0.464	11	0.606
STMN1	5	3	8.92E-47	2.61E-50	0.241	1	0.943
SEPTIN7	10	3	3.73E-45	2.63E-18	0.084	7	0.900
GPI	10	4	9.21E-42	5.47E-11	-0.066	19	0.896
RAB1A	4	3	1.07E-40	1.26E-39	0.166	2	0.953
RBIS	12	3	7.53E-39	1.36E-32	0.196	8	0.764
WSB1	12	1	3.31E-38	3.02E-23	-0.159	17	0.975
ZFYVE16	13	3	3.39E-37	9.17E-19	-0.126	5	0.912
CALCOCO2	19	1	1.44E-35	2.78E-20	0.094	17	0.712
AKAP8L	20	3	1.54E-34	8.89E-06	0.092	19	0.667
IFITM2	10	3	2.34E-34	5.73E-17	-0.198	11	0.873
SNRPN	9	2	1.71E-32	1.73E-22	-0.101	15	0.971
GLUL	7	2	7.12E-32	1.22E-27	0.127	1	0.912
SNX3	4	2	1.97E-31	3.94E-23	-0.150	6	0.948
MYO10	12	4	2.01E-30	9.14E-09	0.100	5	0.943
CLU	9	1	1.41E-29	2.33E-17	-0.050	8	0.973
JTB	5	4	1.69E-29	4.67E-23	-0.197	1	0.950
**SPLICE LBSL cells VS. Control cells**							
MAGED2	8	2	1.07E-116	1.35E-121	0.288	X	0.894
IFITM2	10	6	4.47E-91	3.55E-43	-0.3296	11	0.826
SULF1	16	4	1.82E-68	6.92E-22	0.187	8	0.807
SCOC	4	2	3.90E-62	7.21E-64	0.196	4	0.853
GAPDH	2	2	5.25E-60	0	-0.114	12	0.995
GNAS	21	2	1.41E-56	3.17E-08	0.239	20	0.909

RPL29	4	2	1.04E-54	1.24E-53	0.243	3	0.977
ITM2B	5	2	3.56E-54	2.05E-37	-0.129	13	0.985
RPL9	6	2	3.36E-53	6.84E-34	0.048	4	0.983
GOLGA8B	10	3	1.14E-49	5.84E-48	-0.202	15	0.683
SF3B2	110	4	1.88E-46	1.64E-28	0.317	11	0..844
YWHAZ	15	2	3.18E-44	2.47E-23	0.179	8	0.878
CALM2	15	2	9.97E-42	8.27E-22	0.150	2	0.990
HNRNPC	13	3	7.35E-41	7.59E-15	-0.093	14	0.948
NDUFB5	11	1	7.02E-39	1.42E-37	0.076	3	0.929
RPL17-C18orf32	3	3	2.54E-38	8.16E-39	0.194	18	0.932
EIF4G2	12	2	7.91E-37	1.91E-24	0.165	11	0.954
CKB	7	3	5.21E-36	3.81E-25	-0.266	14	0.905
RPL10	7	2	9.59E-36	1.13E-41	0.188	X	0.958
CYCS	4	2	2.15E-33	3.90E-36	-0.153	7	0.960

Supplemental Table 3. LBSL Associated Differentially Spliced Exons

DSE genes	Chr	5' adjacent exon	DSE exon	3' adjacent exon	cell_coeff	FDR
LGMN.AS2	14	92703807-92704361	92704640-92704707	92709672-92709872	8.724	3.34E-43
PDCD4	10	110871795-110872018	110875966-110876070	110881233-110881535	-3.554	4.97E-15
TARDBP.AS[illegible]	1	11013716-11013965	11016844-11017007	11018733-11018873	4.871	1.79E-12
UBE2V1	20	50081124-50082914	50084129-50084254	50096672-50096820	-3.844	6.88E-12
KARS1	16	75635912-75636098	75636454-75636547	75641564-75641723	3.688	1.29E-10
NDUFS7	19	1383900-1383942	1386518-1386639	1387811-1387847	-5.339	8.41E-09
NASP.AS2	1	45591223-45591270	45606482-45606591	45613169-45613248	2.526	1.41E-07
MIA2	14	39319209-39319291	39320928-39321056	39326864-39327022	-3.392	2.32E-07
HAGH	16	1817168-1817271	1819115-1819223	1819897-1820014	-2.416	5.29E-07
RP11-[illegible]	15	68197440-68197628	68199541-68199681	68205121-68205151	-3.22	5.31E-07
MIF4GD	17	75267538-75267630	75268083-75268192	75270114-75270245	-3.45	7.73E-07
FNTA	8	43056308-43056546	43064101-43064215	43069555-43069659	-3.053	2.52E-06
SRP72	4	56478592-56478649	56483139-56483270	56484736-56484864	2.571	3.40E-06
GALNT10	5	154329572-154329738	154376277-154376462	154380448-154380631	6.409	4.22E-06
COX7B	X	77899440-77899593	77901744-77901867	77902643-77902767	1.636	6.91E-06
PIFO	1	111346910-111347049	111347674-111347772	111348516-111348646	-2.052	7.15E-06
DARS2.AS2	1	173826687-173826786	173828333-173828399	173830660-173830761	-6.781	7.99E-06
NUCB2.AS2	11	17312028-17312120	17315386-17315475	17330127-17330297	-2.757	2.63E-05
EXOC5	14	57239595-57239659	57244165-57244359	57246711-57246858	-4.015	6.46E-05
CLYBL	13	99858861-99859049	99862991-99863092	99864818-99864911	-4.974	9.54E-05

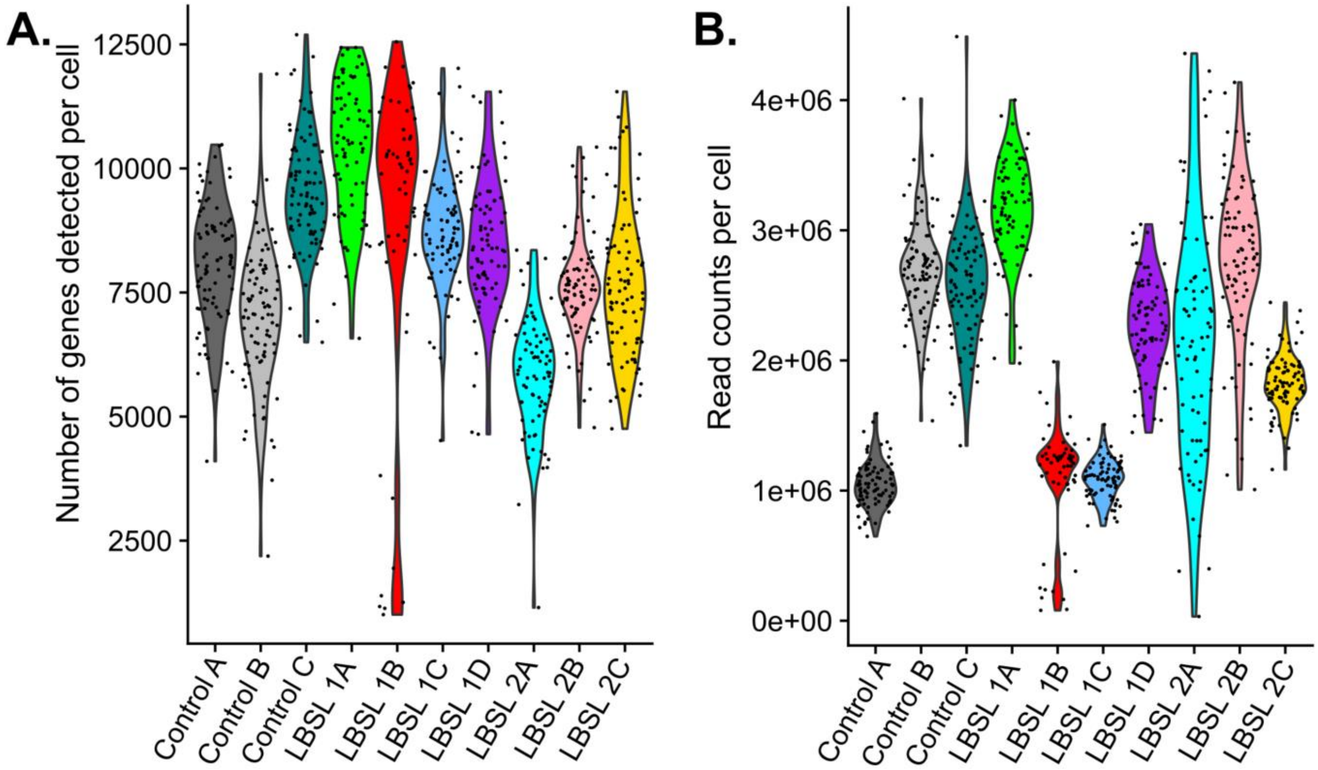

Supplemental Figure 1. Violin plots after quality control. (A) Number of genes detected per cell (> 1000) for each cell line. (B) Read counts per cell indicated by cell line.

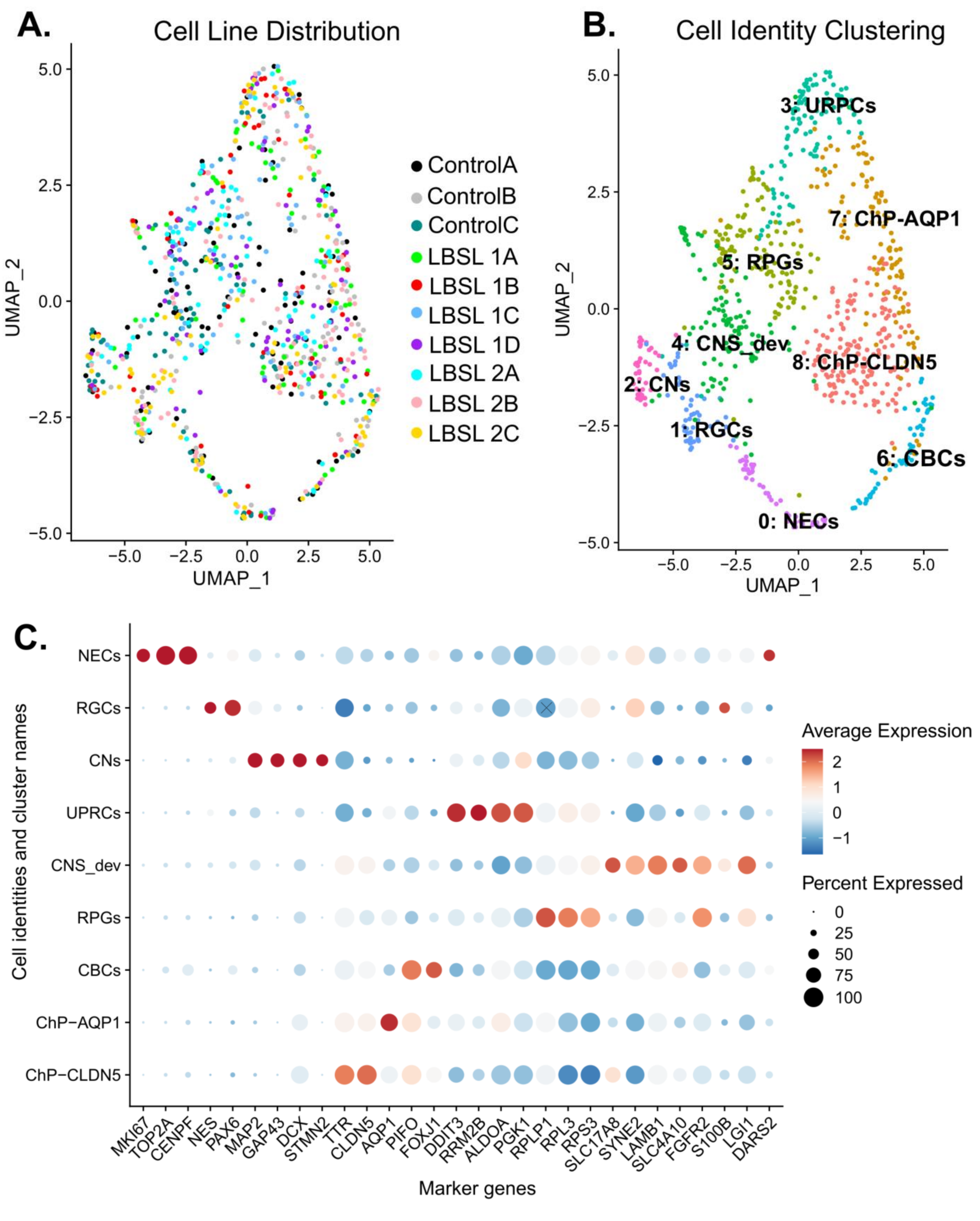

Supplemental Figure 2. Unsupervised clustering and dimension reduction by Uniform Manifold Approximation and Projection (UMAP). (A) Cells from different cell lines were intermixed without an obvious batch effect. (B) Nine transcriptionally distinct clusters were detected. (C)

SUPPLEMENTAL DATA

Dot plot of marker genes to identify each cell cluster. NECs: neuroepithelial cells; RGCs: radial glial cells; CNs: cortical neurons; UPRCs: unfolded protein response-related cells; CNS_dev: cells related to the development of CNS; RPGs: cells highly expressing ribosomal protein genes; CBCs: cilium bearing cells; ChP: choroid plexus.

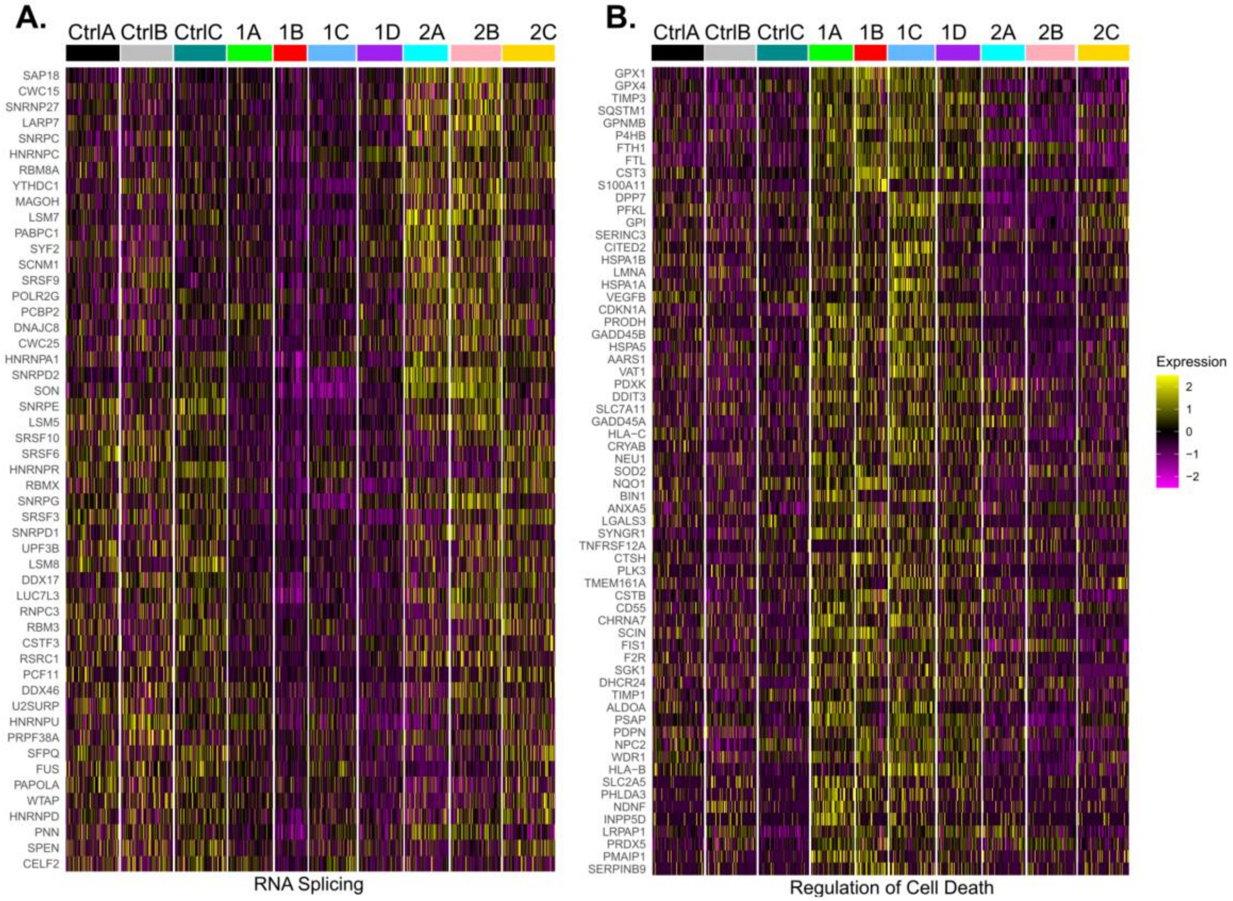

Supplemental Figure 3. Heatmaps of DEGs under the GO terms “RNA splicing” (A) and “Regulation of cell death” (B).

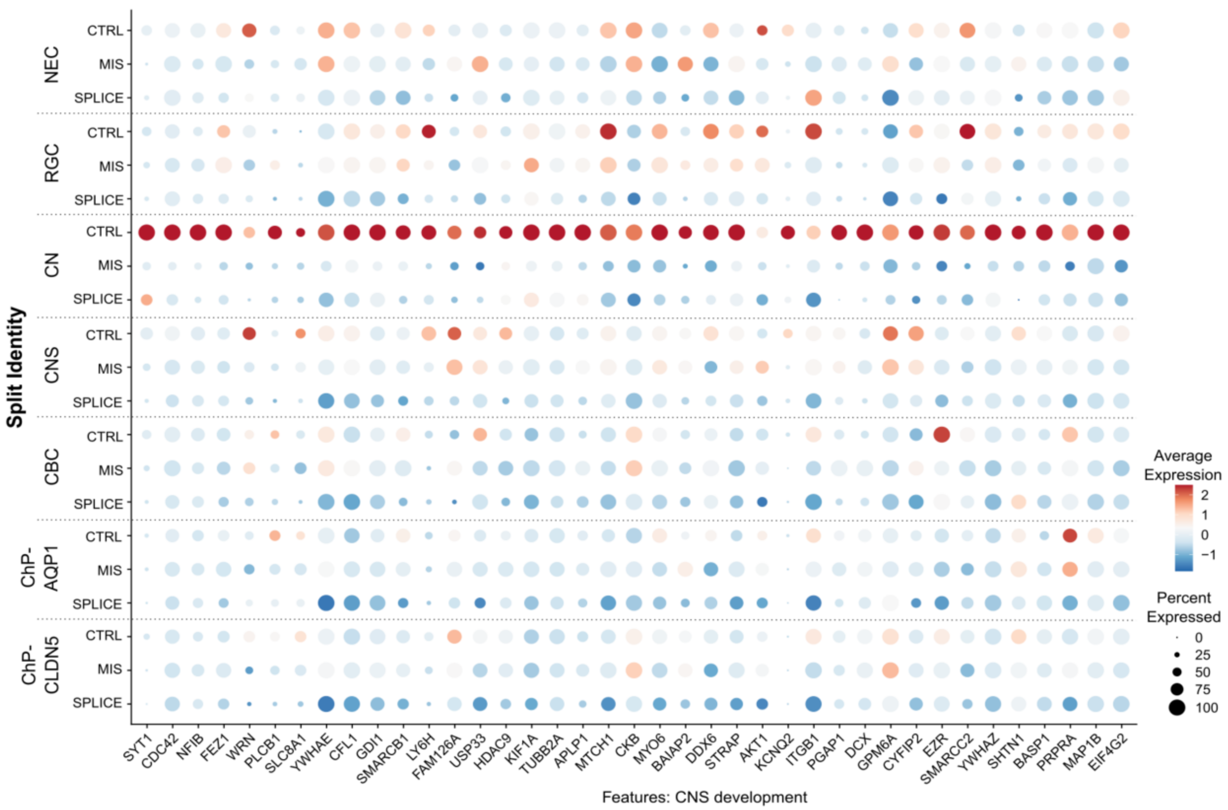

Supplemental Figure 4. Dot plot of DEGs related to CNS development and neuronal differentiation.

LI-COR

Image ID: 0004804_02
Acquire Time: Mar 5, 2020 3:25:19 PM

Page 1

Acquisition Information

#	Image ID	Acquire Time	Channels	Resolution	Intensities	Quality	Analysis	Image Name	Comment
1	0004804_02	Mar 5, 2020 3:25:19 PM	700 800	169um	3.0 3.0	medium	Manual	0004804_02	

Image Display Values

Channel	Color	Minimum	Maximum	K
700	Gray Scale (Black on White)	5.26	6480	0
800	Gray Scale (Black on White)	0.437	6700	0

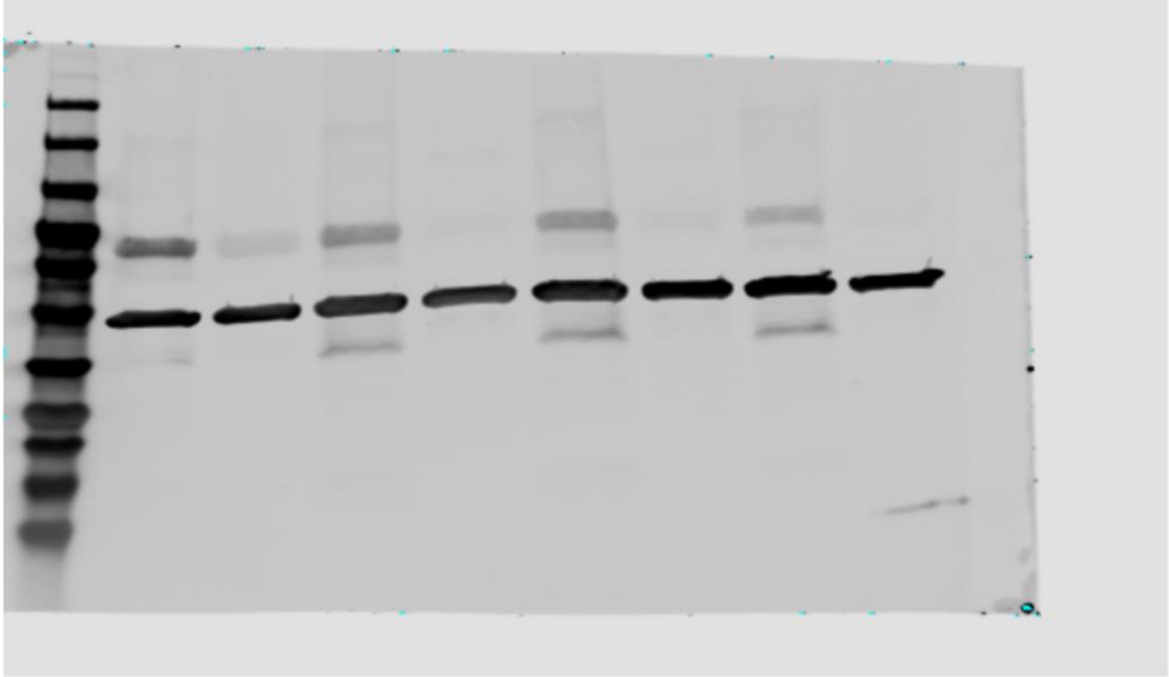

Supplemental Figure 5. LI-COR report of raw western blot data. All samples were run and analyzed from a single gel.
